# Postoperative Opioid Consumption After Discharge: An Update From the Michigan Surgical Quality Collaborative Registry

**DOI:** 10.1097/AS9.0000000000000517

**Published:** 2024-11-07

**Authors:** Ashwin J. Kulkarni, Vidhya Gunaseelan, Chad M. Brummett, Jennifer Waljee, Michael Englesbe, Mark C. Bicket

**Affiliations:** From the * University of Michigan Medical School, Ann Arbor, MI; † Department of Anesthesiology, University of Michigan, Ann Arbor, MI; ‡ Opioid Prescribing Engagement Network, Institute for Healthcare Policy and Innovation, University of Michigan School of Public Health, University of Michigan, Ann Arbor, MI; § Department of Surgery, University of Michigan, Ann Arbor, MI.

**Keywords:** analgesics, consumption, guidelines, health care, opioid, pain, postoperative

## Abstract

**Objective:**

To evaluate opioid consumption for 21 procedures over 4 years from the Michigan Surgical Quality Collaborative (MSQC) registry and update post-discharge prescribing guidelines.

**Background:**

Opioids remain a common treatment for postoperative pain of moderate-to-severe intensity not adequately addressed by nonopioid analgesics, but excessive prescribing correlates with increased usage. This analysis provides updates and compares patient-reported consumption in response to new guidelines.

**Methods:**

We examined data from the MSQC registry for opioid-naive adult patients undergoing surgery between January 1, 2018, and October 31, 2021. The primary outcome was patient-reported opioid consumption in oxycodone 5 mg equivalents. Guidelines were anchored to the 75th percentile of consumption, updating previous guidelines from January 2020 based on data from January 1, 2018, to May 31, 2019.

**Results:**

39,493 opioid-naive surgical patients (average age 53.8 years [SD 16.4], 56.3% female, 19.1% non-White, 43.9% with public insurance) were included. Guidelines did not change for 7 of the 16 procedures including the most common procedures: minor hernia, laparoscopic cholecystectomy, laparoscopic appendectomy, and laparoscopic hysterectomy. Recommended prescribing ranges were lower for 9 procedures, with most (8) procedures having a reduction of 5 pills. Prescribing guidelines were developed for 5 new procedures. All procedures had upper-limit guidelines of 10 pills or less.

**Conclusions:**

For most procedures, patient-reported opioid consumption decreased between 2018 and 2021 when compared to the period between 2018 and 2019. New guidelines were established for a dozen procedures to balance maximizing pain control with reducing harms from inappropriate prescribing.

## INTRODUCTION

Rates of surgical care in the US are increasing, especially among those over 65, with 1 in 9 Americans reporting undergoing surgery.^1^ Despite an initial disruption from the COVID-19 pandemic, surgical volume returned to 2019 levels in nearly all surgical specialties by early 2021.^2^ Post-surgical pain, affecting 20% to 30% of patients, is a common complication that is often managed with opioids.^3^ However, the rise in opioid prescriptions coupled with a lack of evidence-based guidelines for prescribing led to significant public health issues including increased morbidity, mortality, and unused medications.^4,5^ The prevalence of surplus opioids contributes to harm in 2 primary ways: long-term prescription opioid use and misuse.^6–8^ In fact, the fourfold increase in US opioid prescribing from 1999 to 2010 mirrored a roughly fourfold increase in prescription opioid overdose deaths during that time.^9,10^

In response, numerous groups have developed and disseminated opioid prescribing guidelines, which have shown promising results. Following the issuance of Centers for Disease Control and Prevention (CDC) opioid prescribing guidelines, decreases were noted in opioid prescriptions and high-risk prescribing practices (such as concurrent benzodiazepine and opioid prescriptions) coupled with an increase in the utilization of non-opioid pain management approaches for chronic pain.^11–13^ Motivated by the CDC’s success, the Michigan Surgical Quality Collaborative (MSQC), comprising 70 hospitals across Michigan and all major surgical facilities in the state, collaborated with the Michigan Overdose Prevention Engagement Network (OPEN) to unveil 4 sets of guidelines between October 2017 and January 2020^14,15^ for postoperative management based on patient-reported consumption. Subsequently, these guidelines resulted in a 50% decrease in patient-reported opioid consumption. Concurrent with these declines, patients also reported consistent levels of satisfaction and postoperative pain intensity, an important factor considering that patients with moderate or severe pain are 1.8 or 3.0 times more likely to receive an opioid prescription compared with patients with mild pain, respectively.^16^ Although the success of the initial analysis was promising, it focused on 16 procedures and a 2-year sample. While post-surgical prescribing has been noted to decline since 2016, these changes in prescribing were likely to translate into a need to re-examine registry data to ensure guidelines reflect contemporary practice, especially considering the COVID-19 pandemic.^17^ It currently remains unclear whether opioid consumption rates would plateau, suggesting additional reductions may compromise pain control after surgery, or would further decline in response to the implementation of lower prescribing recommendations.

Our goal was to establish updated MSQC prescribing guidelines via 3 aims. First, we reported prescribing trends over 4 years with a larger patient sample size to re-assess consumption on the 16 previously reported procedures. Second, we broadened our analysis to expand prescribing guidelines for 5 additional procedures. Third, we studied patterns of consumption between surgical approaches (ie, open vs laparoscopic) for 4 common surgeries to understand which procedure guidelines may be appropriate to simplify in a combined format. Altogether, this analysis scrutinized whether the favorable patterns in consumption have endured following previous guideline implementation.

## METHODS

The Institutional Review Board of the University of Michigan deemed this study of de-identified secondary data exempt from review and waived the requirement for informed consent. This study follows a preregistered Strengthening the Reporting of Observational Studies in Epidemiology reporting guidelines.^18^ This work was funded by the Michigan Department of Health and Human Services under award E20242492-002.

### Data Source and Study Cohort

The MSQC maintains a validated clinical registry covering general, vascular, and gynecological surgeries, capturing a random sample of 50,000 patients across MSQC’s 70 hospitals annually. Hospital participation is voluntary, with trained clinical nurse reviewers at each hospital to extract patient characteristics, perioperative care processes, and 30-day postoperative follow-up. Cases within the MSQC are randomly selected through an algorithm designed to minimize selection bias and represent all eligible cases within each hospital. Data undergo regular audits to ensure accuracy. Of the 70 hospitals in the MSQC, 69 were able to furnish valid prescription data for this project and were consequently included in the analysis.

In 2016, OPEN was established at the University of Michigan. OPEN represents a collaboration among clinicians across various specialties such as surgery, primary care, and anesthesiology, along with the Michigan Department of Health and Human Services and Blue Cross Blue Shield of Michigan, a prominent private payer within the state. This partnership, driven by clinicians, brings together important stakeholders united in the goal of preventing harm from opioids by enhancing postoperative opioid prescribing practices and patient education.

Previously, 16 procedures had prescription guideline development as they had 25 or more cases with opioid consumption data.^15^ Ultimately, these directives advocated for prescribing an opioid quantity that aligns with or falls below the 75th percentile of patient-reported usage and lead to a 50% reduction in postoperative opioid prescribing.^14,19,20^ Building on this success, our study includes additional years of data to analyze patient-reported consumption across 21 surgeries. With our larger sample, 5 new procedures were added as they had more than 25 cases with opioid consumption data (Table 1).

**TABLE 1.** Inclusion and Exclusion Criteria for Patient Cases

Criteria	Number of Cases Included	Number of Cases Excluded
Surgery in the MSQC between January 1, 2018, and October 31, 2021	150,906	
Ages ≥18older	150,886	20
No history of opioid use	65,468	85,418
Opioid prescription at time of discharge	53,486	11,982
Valid consumption data	40,089	13,397
Surgical procedure of interest (>25 cases)	39,493	596

This analysis included MSQC surgical procedures between January 1, 2018, and October 31, 2021, on adults who were 18 years of age or older. Included patients had to be opioid-naive, which was defined by patient survey questions, except for carotid endarterectomy procedures, for which both opioid-exposed patients were also included. In hospitals participating in this data collection initiative, prescription details were extracted from medical records for a subset of patients enrolled in the MSQC registry. These patients were subsequently contacted to provide patient-reported outcomes, as outlined in greater detail previously.^15^ Patients were surveyed both via telephone and mail on postoperative day 30 and were allotted 90 days to respond. Respondents were asked to report the number of opioid tablets consumed during the initial 30 days following surgery, a time when most opioids are consumed and when patients have shown to accurately report consumption.^21,22^ Patients who did not have discharge opioid prescriptions and those without valid prescription and consumption data were excluded.

### Study Outcomes

The study’s primary outcome was patient-reported opioid consumption for each surgical procedure. Consumption data were converted into oral hydrocodone equivalents (OME) to standardize potency of different medications and allow for comparisons across patients and opioid types. For clarity, consumption data were then reported in terms of the equivalent number of 5 mg oxycodone tablets, with one 5 mg oxycodone tablet equivalent to 7.5 mg OME, verified CDC conversion factors.^11^ Zero consumption statistics were also reported to provide further context on consumption patterns.

Secondary outcomes were the updated prescribing guidelines established by a multidisciplinary team of experts at OPEN based on the 2018–2019 data.^15^ These established the 75th percentile as the upper limit on prescribing guidelines and serve as the analytical benchmark. Opioid consumption amounts and guidelines are either compared to previous guidelines based on 2018–2019 MSQC consumption data when available or reported as novel findings for 5 newly reported procedures. Four surgeries (appendectomy, cholecystectomy, colectomy, and hernia) were further analyzed to assess if distribution and range of opioid consumption differed between procedure approaches (laparoscopic vs open or major vs minor hernia).

### Additional Measures

Characteristics used to define the patient cohort for this analysis (and their response options) include age (in years), sex (male, female, other), race/ethnicity (White non-Hispanic, Black non-Hispanic, Hispanic, other, unknown), insurance type (private, Medicare, Medicaid, Medicare and Medicaid, other/uninsured), and surgical priority (elective or emergent).

### Statistical Analysis

Descriptive statistics were used to examine patient demographics for the overall cohort. Descriptive statistics were also calculated for patient characteristics for each specific procedure to account for variation. Opioid consumption was calculated using the patient-reported number of pills consumed and reported as mean, standard deviation, median, 25th/75th percentile, and interquartile range for each surgical procedure. Zero consumption statistics were provided for each procedure. For the 4 surgeries that had more than 1 type of approach, the Kolmogrov-Smirnov test was used to determine if their consumption patterns differed. This nonparametric and distribution-free test evaluates the null hypothesis of whether 2 random samples appear to have different statistical distributions.^23,24^

For further validation, a sensitivity analysis was performed using a smaller secondary cohort from June 1, 2019, to October 31, 2021. The cohort characteristics, patient-reported consumption, and cohort characteristics by procedure can be found in Supplementary Tables 2–4, http://links.lww.com/AOSO/A428. All study analyses took place after the study period and all statistical tests were performed using Stata version 16 (StataCorp LLC).

## RESULTS

Out of the 150,906 surgeries in the MSQC between January 1, 2018, and October 31, 2021, 39,493 were included. The vast majority, 85,418, were excluded due to a history of opioid consumption with a further 13,397 excluded due to a lack of reported consumption data leading to a response rate of 75.0%. Inclusion and exclusion data by criteria can be found in Table 1.

On average, patients were 53.8 years old (SD 16.4), with 56.3% female, 12.1% non-White, and 43.9% with public insurance. Overall, more patients underwent elective cases (76.3%) than emergent cases (23.7%). Aggregate patient characteristics for the primary cohort are provided in Table 2. Specific patient characteristics for the 4 most common procedures, minor hernia (29.1%), laparoscopic cholecystectomy (25.0%), laparoscopic appendectomy (9.8%), and laparoscopic hysterectomy (9.2%), are found in Table 3. Patient characteristics for the remaining procedures are detailed in Supplementary Table 1, http://links.lww.com/AOSO/A428.

**TABLE 2.** Primary Cohort Demographic Characteristics

Demographic Characteristic	Primary Cohort: N = 39,493	Procedure Type	Primary Cohort: N = 39,493
Age, y		Colectomy – Laparoscopic	1735 (4.4%)
Mean	53.8	Laparoscopic enterolysis	64 (0.2%)
SD	16.4	Laparoscopic closure of enterostomy with resection and anastomosis	55 (0.1%)
**Sex**		Colectomy – Open	1262 (3.2%)
Male	17,264 (43.7%)	Ileostomy/colostomy	379 (1.0%)
Female	22,229 (56.3%)	Open small bowel resection/enterolysis	438 (1.1%)
Race/ethnicity		Anti-reflux (Nissen) – Laparoscopic	609 (1.5%)
White, non-Hispanic	31,946 (80.9%)	Appendectomy – Laparoscopic	3887 (9.8%)
Black, non-Hispanic	3412 (8.6%)	Appendectomy – Open	241 (0.6%)
Hispanic	965 (2.4%)	Cholecystectomy – Laparoscopic	9877 (25.0%)
Other	432 (1.1%)	Cholecystectomy – Open	242 (0.6%)
Unknown	2738 (6.9%)	Minor hernia	11,473 (29.1%)
Insurance type		Major hernia	1604 (4.1%)
Private	21,105 (53.4%)	Thyroidectomy	814 (2.1%)
Medicare	11,020 (27.9%)	Hysterectomy – Abdominal	1202 (3.0%)
Medicaid	5763 (14.6%)	Hysterectomy – Laparoscopic	3651 (9.2%)
Medicare and Medicaid	561 (1.4%)	Hysterectomy – Vaginal	1624 (4.1%)
Other/uninsured	1044 (2.6%)	Carotid endarterectomy	94 (0.2%)
Surgical priority		Excision of rectal tumor, transanal approach	71 (0.2%)
Elective	30,149 (76.3%)	Pancreatectomy	105 (0.3%)
Emergent	9344 (23.7%)	Gastrorrhaphy, suture of perforated duodenal or gastric ulcer, wound, or injury	66 (0.2%)

**TABLE 3.** Primary Cohort Demographic Characteristics of 4 Most Common Procedures

	Minor Hernia	Laparoscopic Cholecystectomy	Laparoscopic Appendectomy	Laparoscopic Hysterectomy
N (%)	11,473 (29.1)	9,877 (25.0)	3,887 (9.8)	3,651 (9.2)
Age, mean (SD), y				
Mean	58.3	50.6	43.3	49.6
SD	15.33	17.3	17.29	12.45
Sex				
Male	9309 (81.1%)	2850 (28.9%)	1774 (45.6%)	0 (0.0%)
Female	2164 (18.9%)	7027 (71.1%)	2113 (54.4%)	3651 (100.0%)
Race/ethnicity				
White, non-Hispanic	9417 (82.1%)	8045 (81.5%)	3213 (82.7%)	2762 (75.7%)
Black, non-Hispanic	858 (7.5%)	790 (8.0%)	230 (5.9%)	406 (11.1%)
Hispanic	190 (1.7%)	336 (3.4%)	154 (4.0%)	76 (2.1%)
Other	101 (0.9%)	121 (1.2%)	53 (1.4%)	42 (1.2%)
Unknown	907 (7.9%)	585 (5.9%)	237 (6.1%)	365 (10.0%)
Insurance type				
Private	5735 (50.0%)	5289 (53.5%)	2493 (64.1%)	2447 (67.0%)
Medicare	3954 (34.5%)	2384 (24.1%)	512 (13.2%)	536 (14.7%)
Medicaid	1365 (11.9%)	1803 (18.3%)	622 (16.0%)	547 (15.0%)
Medicare and Medicaid	142 (1.2%)	136 (1.4%)	26 (0.7%)	51 (1.4%)
Other/uninsured	277 (2.4%)	265 (2.7%)	234 (6.0%)	70 (1.9%)
Surgical priority				
Elective	11,043 (96.3%)	6522 (66.0%)	286 (7.4%)	3640 (99.7%)
Emergent	430 (3.7%)	3355 (34.0%)	3601 (92.6%)	11 (0.3%)

### Patient-Reported Opioid Consumption (Primary Outcome)

**FIGURE 1.**
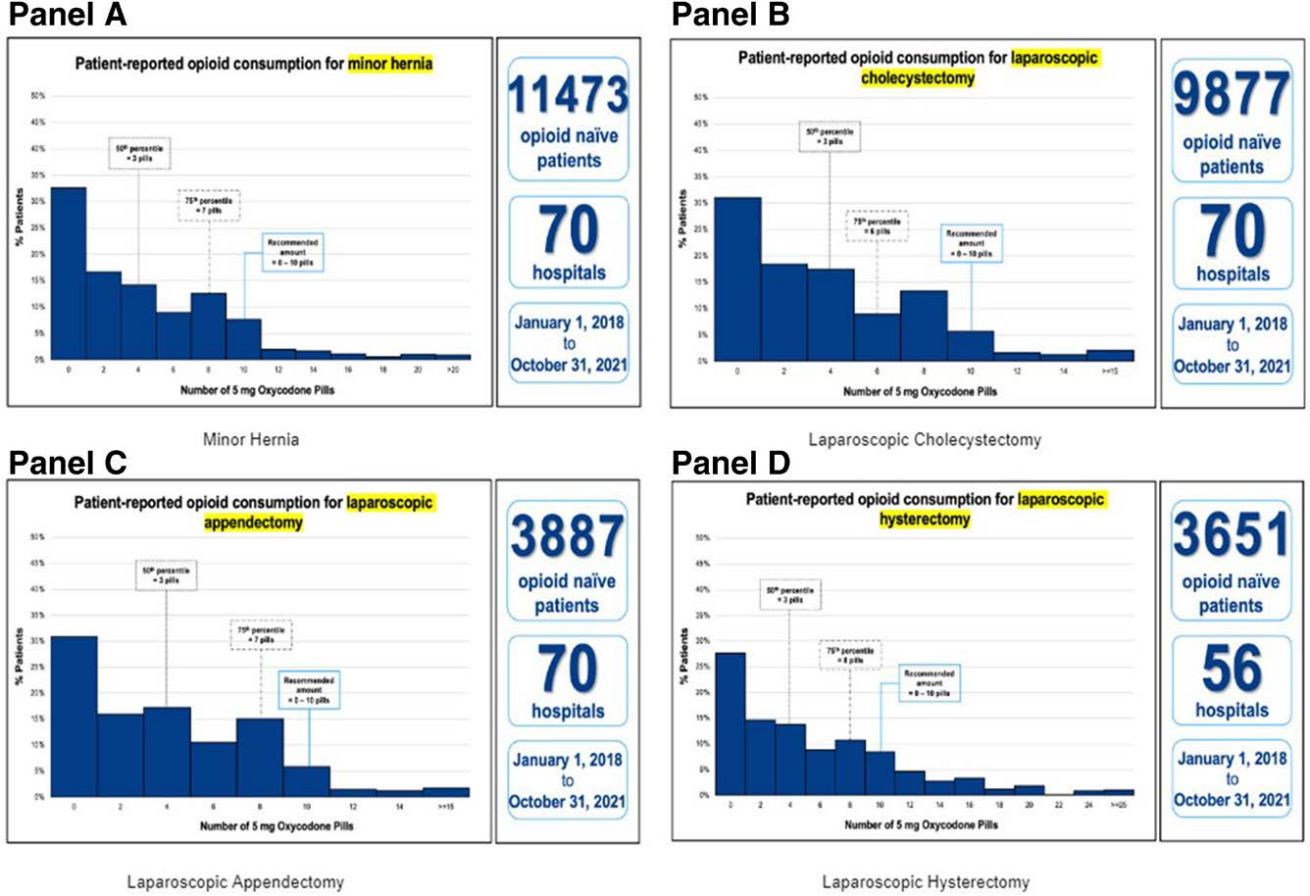
Patient-reported opioid consumption for 4 most frequent procedures. Patient-reported opioid consumption for minor hernia, laparoscopic cholecystectomy, laparoscopic appendectomy, and lap hysterectomy which combined for 73.1% of all procedures. Figures for the remaining 17 procedures can be found in Supplementary Figure 1, http://links.lww.com/AOSO/A428.

For all procedures, mean and median opioid consumption was 4.9 and 2.8 pills, respectively. Mean 25th percentile consumption was 0.1 pills and 75th percentile consumption was 7.6 pills. The 4 procedures with the highest volume (minor hernia, laparoscopic cholecystectomy, laparoscopic appendectomy, and laparoscopic hysterectomy) had mean and median consumption of 4.2 and 2.8 pills with the 25th and 75th percentiles reported as 0 and 6.8 pills, respectively. Five procedures without previous prescribing guidelines were reported in our study: pancreatectomy (0.3%), transanal excision of rectal tumor (0.2%), laparoscopic enterolysis (0.2%), laparoscopic enterostomy closure (0.2%), and gastrorrhaphy (0.1%). These procedures had a 5.4 pill mean and a 3.2 pill median along with 25th and 75th percentiles of 0.3 and 8.1 pills, respectively. Graphs of patient-reported opioid consumption for the 4 most common procedures can be found in Figure 1, with consumption graphs of all procedures largely following the right-skewed distribution seen. Furthermore, Supplementary Figure 1, http://links.lww.com/AOSO/A428, details consumption for all procedures with Table 4 summarizing total opioid prescribing data. Cohort characteristics were then stratified by procedure type with results available in Supplementary Table 1, http://links.lww.com/AOSO/A428. Furthermore, updated guidelines and histograms can be found on michigan-open.org/prescribing-recommendations/.

**TABLE 4.** Opioid Consumption* by Procedure for Primary Cohort

Surgical Procedures	Primary Cohort (January 1, 2018 to October 31, 2021)							
	N	Mean	SD	Median	25th Percentile	75th Percentile	IQR	% Cases With Zero Consumption
Current procedures								
Colectomy – Laparoscopic	1735	4.5	6.8	1.3	0.0	6.7	6.7	42.6
Colectomy – Open	1262	6.2	8.4	3.3	0.0	10.0	10.0	35.1
Ileostomy/colostomy	379	6.2	8.1	4.0	0.0	10.0	10.0	34.6
Open small bowel resection/enterolysis	438	4.9	6.5	2.0	0.0	8.0	8.0	40.6
Anti-reflux (Nissen) – Laparoscopic	609	3.8	5.8	1.3	0.0	5.3	5.3	37.3
Appendectomy – Laparoscopic	3887	3.7	4.3	2.7	0.0	6.7	6.7	30.9
Appendectomy – Open	241	3.9	4.9	2.0	0.0	6.7	6.7	37.8
Cholecystectomy – Laparoscopic	9877	3.7	4.5	2.7	0.0	6.0	6.0	30.9
Cholecystectomy – Open	242	6.0	7.1	4.0	0.0	10.0	10.0	35.1
Minor hernia	11473	4.1	5.1	2.7	0.0	6.7	6.7	32.6
Major hernia	1604	5.2	6.4	3.3	0.0	8.0	8.0	27.2
Thyroidectomy	814	2.8	5.1	1.0	0.0	3.3	3.3	44.0
Hysterectomy – Abdominal	1202	7.4	8.3	5.3	0.7	12.0	11.3	24.0
Hysterectomy – Laparoscopic	3651	5.4	6.5	3.3	0.0	8.0	8.0	27.5
Hysterectomy – Vaginal	1624	5.0	5.6	3.0	0.0	8.0	8.0	30.7
Carotid endarterectomy	94	4.0	13.0	0.9	0.0	4.7	4.7	46.8
New procedures								
Pancreatectomy	105	8.6	16.9	3.3	0.0	10.0	10.0	37.1
Excision of rectal tumor, transanal approach	71	2.7	4.4	0.0	0.0	5.3	5.3	50.7
Gastrorrhaphy, suture of perforated duodenal or gastric ulcer, wound, or injury	66	7.1	7.1	6.7	1.3	10.0	8.7	19.7
Laparoscopic enterolysis	64	3.2	3.8	2.0	0.0	5.1	5.1	37.5
Laparoscopic closure of enterostomy with resection and anastomosis	55	5.4	6.2	4.0	0.0	10.0	10.0	34.5

* Consumption reported in 5 mg oxycodone equivalents. Some procedures have no reported current recommendations as previous prescription data was not available.

IQR indicates interquartile range.

### Previous Versus Updated Opioid Prescribing Guidelines (Secondary Outcome)

Examining consumption data, 9 of 16 procedures had prescribing guidelines that were lower than the previous 2019 guidelines: open colectomy, ileostomy/colostomy, open small bowel resection, laparoscopic Nissen fundoplication, open cholecystectomy, abdominal hysterectomy, laparoscopic hysterectomy, vaginal, hysterectomy, and carotid endarterectomy. The upper limit of these guidelines all had a 5-pill reduction, except for abdominal hysterectomy which had a reduction of 10 pills. Meanwhile, the 5 newly reported procedures had prescription guidelines with upper ranges of 5 or 10 pills. Overall, patient-reported opioid consumption appeared to decline for the 2018 to 2021 period when compared to 2017 to 2019, from which the previous guidelines were based. Further information on prescribing guidelines is detailed in Table 5.

**TABLE 5.** Opioid Prescribing Guidelines by Specific Procedure

Surgical Procedures	Surgical Volume (%)	Opioid Pills		
		Previous Guidelines*	Revised Guidelines	75th Percentile Consumption
Current procedures				
Colectomy – Laparoscopic	4.4	0–10	No change	6.7
Colectomy – Open	3.2	0–15	0–10	10.0
Ileostomy/colostomy creation, re-siting, or closure	0.1	0–15	0–10	10.0
Open small bowel resection or enterolysis	1.1	0–15	0–10	8.0
Anti-reflux (Nissen) – Laparoscopic	1.5	0–10	0–5	5.3
Appendectomy – Laparoscopic	9.8	0–10	No change	6.7
Appendectomy – Open	0.1	0–10	No change	6.7
Cholecystectomy – Laparoscopic	25.0	0–10	No change	6.0
Cholecystectomy – Open	0.1	0–15	0–10	10.0
Minor hernia	29.1	0–10	No change	6.7
Major hernia	4.1	0–10	No change	8.0
Thyroidectomy	2.1	0–5	No change	3.3
Hysterectomy – Abdominal	3.0	0–20	0–10	12.0
Hysterectomy – Laparoscopic	9.2	0–15	0–10	8.0
Hysterectomy – Vaginal	4.1	0–15	0–10	8.0
Carotid endarterectomy	0.1	0–10	0–5	4.7
New procedures†				
Pancreatectomy	0.3	None	0–10	10.0
Excision of rectal tumor, transanal approach	0.2	None	0–5	5.3
Gastrorrhaphy, suture of perforated duodenal or gastric ulcer, wound, or injury	0.2	None	0–10	10.0
Laparoscopic enterolysis	0.1	None	0–5	5.1
Laparoscopic closure of enterostomy (large of small bowel) with resection and anastomosis)	0.1	None	0–10	10.0

* Previous recommendations based on MSQC Medicare claims (Parts A, B, D) from January 1, 2018, to May 31, 2019, as outlined in Brown et al.^14^

† No previous recommendations have been established for these 5 procedures.

### Patient-Reported Opioid Consumption by Surgery Type

When comparing the distribution of opioid consumption, 4 types of procedures were analyzed. Laparoscopic versus open procedures were compared for cholecystectomy, appendectomy, and colectomy while minor versus major repair for hernia was compared. The consumption patterns were found to be similar for laparoscopic versus open appendectomies (*P* = 0.10). On the other hand, opioid consumption patterns differed for laparoscopic versus open colectomies (*P* < 0.01), laparoscopic versus open cholecystectomies (*P* < 0.01), and major versus minor hernia repairs (*P* < 0.01), where open and major procedures had higher consumption (Table 6).

**TABLE 6.** Kolmogorov-Smirnov Analysis for Opioid Consumption by Surgery Type

Group	Comparison	Primary Cohort (January 1, 2018 to October 31, 2021)	
		Kolmogorov-Smirnov Test Statistic	*P*
Appendectomy	Laparoscopic vs open	0.081	0.100
Cholecystectomy	Laparoscopic vs open	0.191	<0.001
Colectomy	Laparoscopic vs open	0.111	<0.001
Hernia	Major vs minor	0.077	<0.001

### Sensitivity Analysis of the Primary Cohort

In a sensitivity analysis using a modified secondary cohort, a total of 28,721 patients from June 1, 2019, to October 31, 2021, were included. Within this cohort, patient-reported opioid consumption was reported with a mean of 4.6 pills, a median of 2.6 pills, and 25th and 75th percentile consumption of 0.0 and 7.1 pills, respectively. Findings were unchanged for prescribing guidelines and Kolmogorov-Smirnov analysis of patterns of consumption (Supplementary Tables 2–5, http://links.lww.com/AOSO/A428).

## DISCUSSION

Our study provides a detailed analysis of the state of Michigan’s patient-reported opioid consumption and the resulting updated prescribing guidelines. All procedures had upper-limit guidelines of 10 pills or less. Three major findings are of particular importance: most procedures identify declining patient-reported opioid consumption, 9 procedures have revised guidelines with a mean upper-limit reduction of 5.6 pills, and appendectomies, whether performed open or laparoscopic, can be combined into one category given similarities in consumption patterns.

Our findings, validated by decreased patient-reported consumption patterns, show a promising effect of the increased emphasis on opioid stewardship, which some have defined as the appropriate use of opioids to maximize pain relief and minimize adverse events and which served as the original motivating factor behind OPEN’s opioid prescribing guidelines.^25–29^ Overall, the registry captures data from sites with variation in approaches to opioid stewardship which both increases external validity due to mimicking of real-life practice while decreasing internal validity due to variation in practice styles. Nevertheless, these trends are reaffirmed when assessing our primary and secondary outcomes: patient-reported consumption and updated guidelines. Reductions in patient-reported consumption allowed an expert multidisciplinary team to revise previous guidelines, with changes of reductions in suggested ranges for 9 of 16 procedures. Furthermore, of the 4 most frequent procedures, only laparoscopic hysterectomy noted a reduction in updated guidelines, pointing toward stable patterns of prescribing and consumption among common procedures, while more uncertainty exists among less frequently performed operations. Overall, this decrease in patient-reported opioid consumption and the updated guidelines which followed is encouraging considering previous literature showing that responsible prescribing does not lead to worsening patient-reported outcomes.^30–32^ This is especially welcome considering previous literature reporting that even though patients use just 27% of opioids prescribed to them, each additional prescribed pill was correlated with a 0.53 pill increase in consumption.^15^ That said, it is important to remember that the guidelines offer recommended amounts for most, but not all, patients given that they are anchored to the 75th percentile of consumption. There will of course be patients who have unique or special needs for pain management, which may necessitate more frequent monitoring and follow-up visits after surgery, an increased number of refills in the post-discharge period, or higher doses of opioids to sufficiently treat pain after surgery. We encourage providers to consider holistic factors when managing a patient’s postoperative pain as these guidelines may not capture the experiences of patients with complex pain or significant opioid exposure, who may require additional options and closer postoperative monitoring to ensure appropriate pain management.

While the effects of opioid stewardship remain clear, there is more nuance regarding guidelines for a particular procedure: abdominal hysterectomies. This was the only procedure where the range of pills within the updated guideline (0–10 pills) remained below the 75th percentile for consumption (12 pills). Abdominal hysterectomies possessed the largest interquartile range of all procedures at more than 11 pills, the second-highest mean of 7 pills, and the second-lowest percent of zero consumption patients at 20%. Altogether, consumption patterns for abdominal hysterectomies displayed a rightward skew (Supplementary Figure 1, http://links.lww.com/AOSO/A428), which may partially account for the 2-pill difference in guidelines compared with 75th percentile. Furthermore, the sensitivity analysis featuring the secondary cohort reported a 75th percentile consumption of 10 pills, further supporting the lower updated guideline range of 0 to 10 pills.

After reporting patient-reported opioid consumption by procedure, this analysis also assessed differences in consumption patterns by procedure type. The Klomogorov-Smirnov statistical distribution assessment found no difference in consumption patterns for laparoscopic versus open appendectomies though differences were identified for laparoscopic versus open cholecystectomies, laparoscopic versus open colectomies, and minor versus major hernia repairs. Despite this, updated guidelines report 0 to 10 pills for each of these procedures. This information appears contradictory on the surface—identical guidelines yet different distributions—but a more careful examination helps to clarify this finding. The guidelines suggest a number of pills to prescribe, which for these procedures is the same range. While the overall range of pills is the same, the pattern of consumption within this range differs for each of the 3 procedure types. Ultimately, even though prescribing guidelines remain similar for these procedures, the analysis identified differing consumption patterns and suggests that hernia repairs, cholecystectomies, and colectomies should be separated based on surgical approach, while open and laparoscopic appendectomies can be combined in future investigations and analyses.

This study is not without its limitations. First, this study relied on patient-reported consumption data which may be subject to social desirability or recall bias, while validation tools such as pill counts or medication diaries remain difficult to implement within a large registry tracking real-world data.^33^ To limit this, data collection took place at postoperative day 30 to decrease the time between their consumption and reporting, a validated timeline used in previous analyses and when the bulk of postoperative consumption occurs and when patients have shown to accurately self-report opioid consumption.^15,21,22^ Consumption may also vary in meaningful ways from prescribing patterns of providers, though the two have been shown to correlate in past studies. An extra pill prescribed remains the strongest risk factor for increased consumption while consumption has shown to mirror prescriptions.^15,34^ Second, this analysis focuses on opioid-naive patients. Preoperative opioid use remains a topic of interest because opioid-exposed patients may have different patterns of consumption, refills, and unique patient management needs. Previous research has correlated preoperative opioid use with increased postoperative opioid consumption compared to opioid-naive counterparts.^35^ Despite this, the inclusion of a wide and representative sample of patients offers a useful framework for opioid prescribing guidelines since this captures the reported outcomes for a broad sample of adults. Third, this analysis lacks other relevant patient-reported measures such as pain intensity, satisfaction, and non-opioid analgesic use such as acetaminophen and non-steroidal anti-inflammatory drugs, which have the potential to confer similar or superior analgesic relief in some acute pain settings.^36^ Nevertheless, it has been noted that patient-reported outcomes have remained consistent following the implementation of guidelines that led to reductions in opioid prescribing.^14,15,37^ Lastly, this analysis relied on data up until October 2021, which includes time periods before and after the COVID-19 pandemic that may have changed consumption patterns. For that reason, we conducted a sensitivity analysis with a narrowed timeframe to validate our findings in the primary cohort.

To our knowledge, this is the first study publishing statewide opioid consumption data which is then used to establish opioid prescribing guidelines in a systematic manner. This provides valuable information to all parties within the healthcare ecosystem. Policymakers and health systems can use this data as further evidence of the effectiveness of opioid prescribing guidelines, allowing for further resource allocation to collect data and update guidelines on a consistent basis. Surgeons, clinical leaders, and patients can view this data as a validation of their efforts to decrease prescribing and consumption while better using nonopioid and nonpharmacological therapies for postoperative pain which have continued to grow with respect to evidence and demonstration of value in accelerating recovery after surgery.^38,39^

## CONCLUSION

Opioid prescribing guidelines continue to play a crucial part in decreasing patient-reported postoperative opioid consumption. Due to this reduction, we established a mean upper-limit reduction of 5.6 pills in 9 of 16 MSQC while also gathering data allowing for guideline establishment for 5 new procedures with a mean upper limit of 7 pills. All procedures had upper-limit guidelines of 10 pills or less. These results suggest that prescribing guidelines continue to serve a vital role in avoiding inappropriate prescribing, which may contribute to poor outcomes ranging from new persistent opioid use to opioid use disorder, while promoting adequate pain control to promote the best outcomes for surgical patients in a safe manner.

## Supplementary Material
