# Supplementary material for: Postoperative Opioid Consumption After Discharge: An Update From the Michigan Surgical Quality Collaborative Registry
Source: Ann Surg Open. 2024 Nov 7;5(4):e517. doi: 10.1097/AS9.0000000000000517 (PMC11661719; doi:10.1097/AS9.0000000000000517)
Supplement: Supplementary file 1 [file as9-5-e517-s001.pdf]

Supplementary Figure 1 Title
Patient-reported opioid consumption by procedure type

Supplementary Figure 1 Caption
Patient-reported opioid consumption and MSQC guidelines for all surveyed procedures.

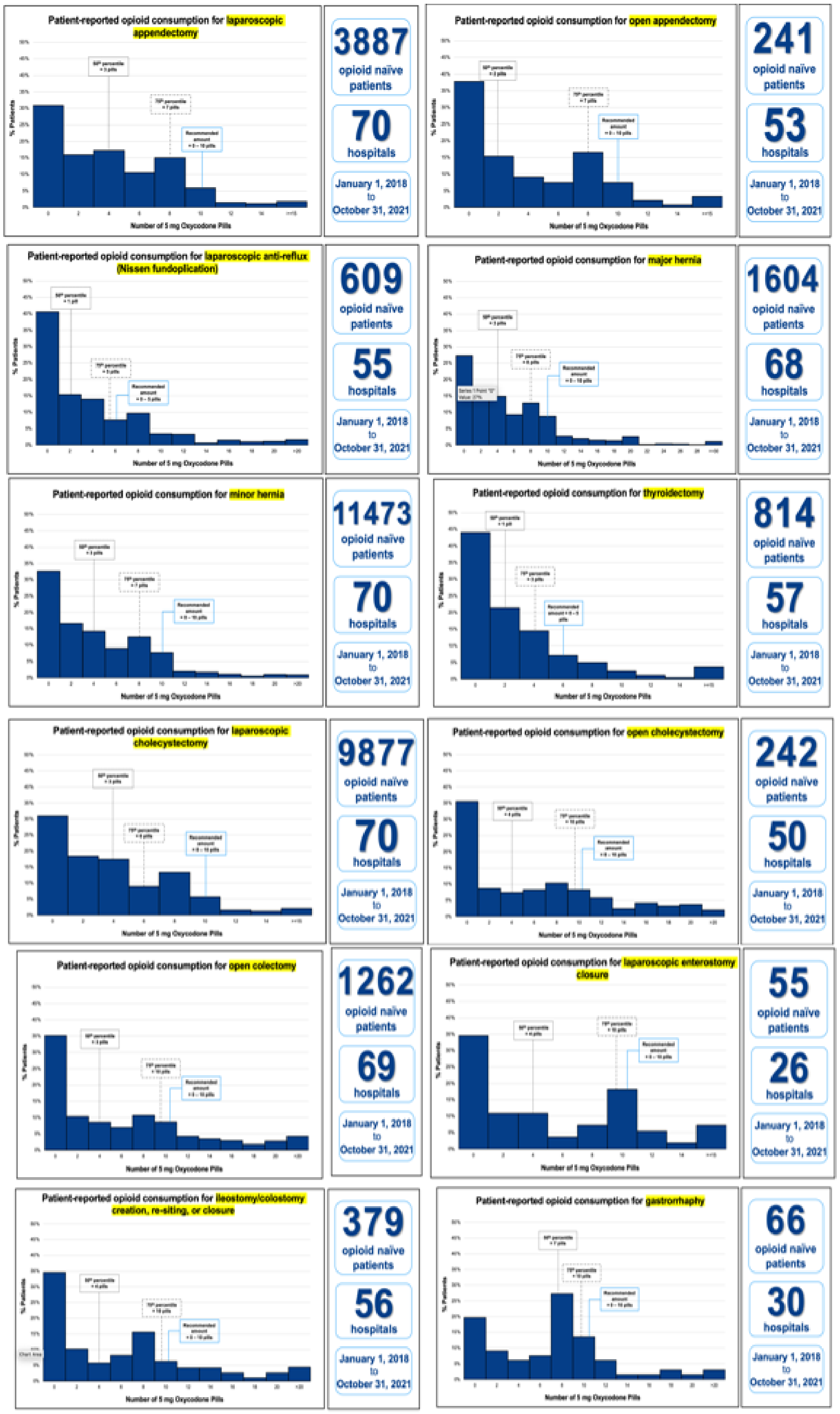

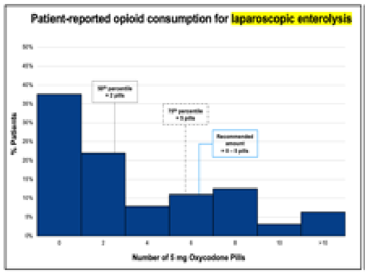

64
opioid naïve patients

31
hospitals

January 1, 2018 to October 31, 2021

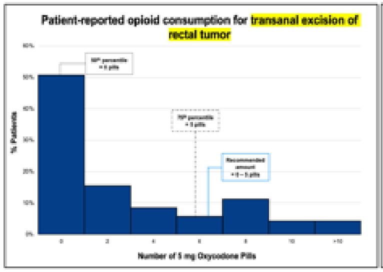

71
opioid naïve patients

19
hospitals

January 1, 2018 to October 31, 2021

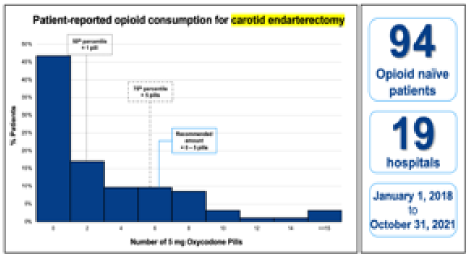

94
Opioid naïve patients

19
hospitals

January 1, 2018 to October 31, 2021

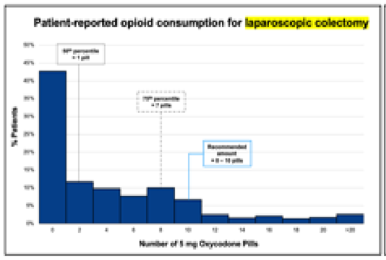

1735
opioid naïve patients

65
hospitals

January 1, 2018 to October 31, 2021

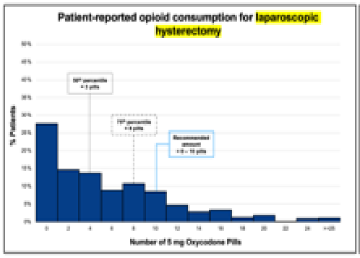

3651
opioid naïve patients

56
hospitals

January 1, 2018 to October 31, 2021

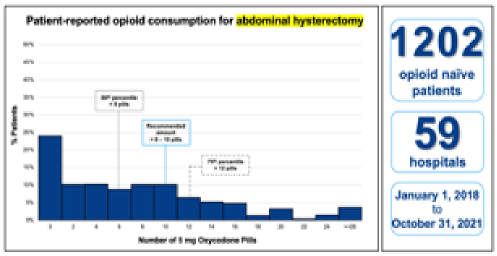

1202
opioid naïve patients

59
hospitals

January 1, 2018 to October 31, 2021

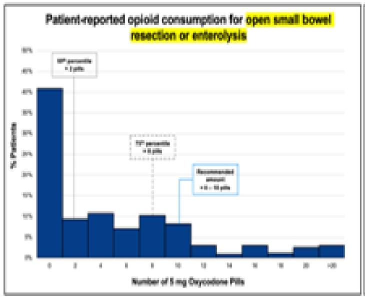

438
opioid naïve patients

58
hospitals

January 1, 2018 to October 31, 2021

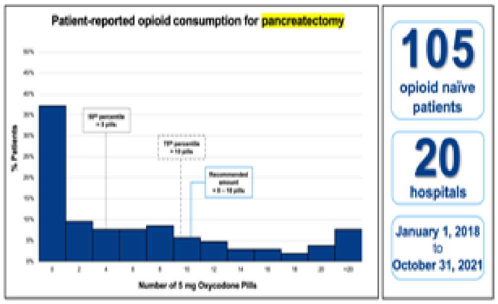

105
opioid naïve patients

20
hospitals

January 1, 2018 to October 31, 2021

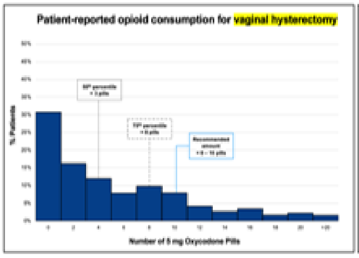

1624
opioid naïve patients

60
hospitals

January 1, 2018 to October 31, 2021

Supplementary Table 1. Patient characteristics by procedure for primary cohort

	**Colectomy - Laparoscopic**	**Colectomy - Open**	**Ileostomy/Colostomy**	**Open Small Bowel Resection/ Enterolysis**	**Anti-reflux (Nissen) - Laparoscopic**	**Appendectomy - Open**	**Cholecystectomy - Open**
	N=1,735	N=1,262	N=379	N=438	N=609	N=241	N=242
**Age, mean (SD) years**							
	62.23(12.56)	62.77 (13.15)	60.84 (13.14)	63.28 (14.86)	61.19 (13.30)	49.18 (18.10)	61.32 (15.75)
**Sex**							
Male	827 (47.7%)	573 (45.4%)	196 (51.7%)	188 (42.9%)	160 (26.3%)	124 (51.5%)	132 (54.5%)
Female	908 (52.3%)	689 (54.6%)	183 (48.3%)	250 (57.1%)	449 (73.7%)	117 (48.5%)	110 (45.5%)
**Race/ethnicity**							
White, non-Hispanic	1,441 (83.1%)	1,087 (86.1%)	317 (83.6%)	369 (84.2%)	538 (88.3%)	188 (78.0%)	184 (76.0%)
Black, non-Hispanic	154 (8.9%)	99 (7.8%)	40 (10.6%)	40 (9.1%)	25 (4.1%)	21 (8.7%)	42 (17.4%)
Hispanic	32 (1.8%)	14 (1.1%)	6 (1.6%)	7 (1.6%)	8 (1.3%)	12 (5.0%)	3 (1.2%)
Other	10 (0.6%)	4 (0.3%)	1 (0.3%)	4 (0.9%)	5 (0.8%)	7 (2.9%)	4 (1.7%)
Unknown	98 (5.6%)	58 (4.6%)	15 (4.0%)	18 (4.1%)	33 (5.4%)	13 (5.4%)	9 (3.7%)
**Insurance type**							
Private	799 (46.1%)	468 (37.1%)	134 (35.4%)	169 (38.6%)	246 (40.4%)	130 (53.9%)	77 (31.8%)
Medicare	737 (42.5%)	598 (47.4%)	162 (42.7%)	214 (48.9%)	281 (46.1%)	53 (22.0%)	113 (46.7%)
Medicaid	143 (8.2%)	126 (10.0%)	69 (18.2%)	35 (8.0%)	64 (10.5%)	39 (16.2%)	42 (17.4%)
Medicare and Medicaid	28 (1.6%)	33 (2.6%)	10 (2.6%)	11 (2.5%)	11 (1.8%)	0 (0.0%)	6 (2.5%)
Other/Uninsured	28 (1.6%)	37 (2.9%)	4 (1.1%)	9 (2.1%)	7 (1.1%)	19 (7.9%)	4 (1.7%)
**Surgical priority**							
Elective	1,551 (89.4%)	628 (49.8%)	343 (90.5%)	75 (17.1%)	591 (97.0%)	19 (7.9%)	101 (41.7%)

Emergent/urgent	184 (10.6%)	634 (50.2%)	36 (9.5%)	363 (82.9%)	18 (3.0%)	222 (92.1%)	141 (58.3%)

	Major Hernia	Thyroidectomy	Hysterectomy - Abdominal	Hysterectomy - Vaginal	Carotid Endarterectomy	Pancreatectomy	Excision of rectal tumor, transanal approach	Gastrorrhaphy, suture of perforated duodenal or gastric ulcer, wound, or injury	Laparoscopic enterolysis	Laparoscopic closure of enterostomy w/ resection and anastomosis
	N=1,604	N=814	N=1,202	N=1,624	N=94	N=105	N=71	N=66	N=64	N=55
**Age, mean (SD) years**										
	57.46 (14.54)	52.41 (15.05)	49.82 (10.89)	51.41 (13.29)	71.61 (8.18)	63.97 (10.33)	65.42 (11.68)	56.50 (15.37)	59.97 (13.77)	56.85 (15.52)
**Sex**										
Male	753 (46.9%)	163 (20.0%)	0 (0.0%)	0 (0.0%)	56 (59.6%)	55 (52.4%)	29 (40.8%)	26 (39.4%)	23 (35.9%)	26 (47.3%)
Female	851 (53.1%)	651 (80.0%)	1,202 (100.0%)	1,624 (100.0%)	38 (40.4%)	50 (47.6%)	42 (59.2%)	40 (60.6%)	41 (64.1%)	29 (52.7%)
**Race/ethnicity**										
White, non-Hispanic	1,301 (81.1%)	607 (74.6%)	755 (62.8%)	1,347 (82.9%)	75 (79.8%)	84 (80.0%)	63 (88.7%)	54 (81.8%)	51 (79.7%)	48 (87.3%)
Black, non-Hispanic	149 (9.3%)	101 (12.4%)	295 (24.5%)	113 (7.0%)	13 (13.8%)	13 (12.4%)	5 (7.0%)	7 (10.6%)	7 (10.9%)	4 (7.3%)
Hispanic	41 (2.6%)	15 (1.8%)	25 (2.1%)	44 (2.7%)	0 (0.0%)	0 (0.0%)	0 (0.0%)	0 (0.0%)	2 (3.1%)	0 (0.0%)
Other	19 (1.2%)	19 (2.3%)	23 (1.9%)	15 (0.9%)	0 (0.0%)	3 (2.9%)	0 (0.0%)	1 (1.5%)	0 (0.0%)	0 (0.0%)
Unknown	94 (5.9%)	72 (8.8%)	104 (8.7%)	105 (6.5%)	6 (6.4%)	5 (4.8%)	3 (4.2%)	4 (6.1%)	4 (6.3%)	3 (5.5%)
**Insurance type**										
Private	763 (47.6%)	452 (55.5%)	777 (64.6%)	969 (59.7%)	12 (12.8%)	49 (46.7%)	31 (43.7%)	16 (24.2%)	27 (42.2%)	22 (40.0%)

Medicare	548 (34.2%)	199 (24.4%)	162 (13.5%)	339 (20.9%)	72 (76.6%)	45 (42.9%)	38 (53.5%)	23 (34.8%)	28 (43.8%)	22 (40.0%)
Medicaid	229 (14.3%)	138 (17.0%)	214 (17.8%)	275 (16.9%)	4 (4.3%)	10 (9.5%)	2 (2.8%)	23 (34.8%)	5 (7.8%)	8 (14.5%)
Medicare and Medicaid	37 (2.3%)	13 (1.6%)	27 (2.2%)	17 (1.0%)	6 (6.4%)	1 (1.0%)	0 (0.0%)	1 (1.5%)	3 (4.7%)	2 (3.6%)
Other/Uninsured	27 (1.7%)	12 (1.5%)	22 (1.8%)	24 (1.5%)	0 (0.0%)	0 (0.0%)	0 (0.0%)	3 (4.5%)	1 (1.6%)	1 (1.8%)
**Surgical priority**										
Elective	1,444 (90.0%)	812 (99.8%)	1,157 (96.3%)	1,614 (99.4%)	82 (87.2%)	101 (96.2%)	70 (98.6%)	0 (0.0%)	15 (23.4%)	55 (100.0%)
Emergent/urgent	160 (10.0%)	2 (0.2%)	45 (3.7%)	10 (0.6%)	12 (12.8%)	4 (3.8%)	1 (1.4%)	66 (100.0%)	49 (76.6%)	0 (0.0%)

Supplementary Table 2. Secondary Cohort Characteristics

Demographic Characteristics	6/1/2019 - 10/31/2021
	**N=28,721**
**Age, mean (SD) years**	53.9 (16.40)
**Sex**	
Male	12,476 (43.4%)
Female	16,245 (56.6%)
**Race/ethnicity**	
White, non-Hispanic	23,054 (80.3%)
Black, non-Hispanic	2,573 (9.0%)
Hispanic	670 (2.3%)
Other	327 (1.1%)
Unknown	2,097 (7.3%)
**Insurance type**	
Private	15,280 (53.2%)
Medicare	8,105 (28.2%)
Medicaid	4,184 (14.6%)
Medicare and Medicaid	420 (1.5%)
Other/Uninsured	732 (2.5%)
**Procedure type**	
Colectomy - Laparoscopic	1,321 (4.6%)
Colectomy - Open	911 (3.2%)
Ileostomy/Colostomy Creation, Re-siting, or Closure	294 (1.0%)
Open Small Bowel Resection or Enterolysis	348 (1.2%)
Anti-reflux (Nissen) - Laparoscopic	522 (1.8%)
Appendectomy - Laparoscopic	2,930 (10.2%)

Appendectomy - Open	176 (0.6%)
Cholecystectomy - Laparoscopic	7,087 (24.7%)
Cholecystectomy - Open	178 (0.6%)
Minor Hernia	8,166 (28.4%)
Major Hernia	1,140 (4.0%)
Thyroidectomy	659 (2.3%)
Hysterectomy - Abdominal	831 (2.9%)
Hysterectomy - Laparoscopic	2,643 (9.2%)
Hysterectomy - Vaginal	1,148 (4.0%)
Carotid Endarterectomy	72 (0.3%)
Excision of rectal tumor, transanal approach	55 (0.2%)
Pancreatectomy	89 (0.3%)
Gastrorrhaphy, suture of perforated duodenal or gastric ulcer, wound, or injury	53 (0.2%)
Laparoscopic enterolysis	49 (0.2%)
Laparoscopic closure of enterostomy (large of small bowel) w/ resection and anastomosis)	49 (0.2%)
**Surgical priority**	
Elective	21,775 (75.8%)
Emergent/urgent	6,946 (24.2%)

Supplementary Table 3. Opioid consumption [A] by procedure for secondary cohort

Surgical procedures	Secondary Cohort (6/1/2019 - 10/31/2021)							
	Number of cases	Mean	SD	Median	25th percentile	75th percentile	IQR	% cases with zero consumption
**Current Procedures**								
Colectomy - Laparoscopic	1321	4.1	6.0	1.3	0.0	6.7	6.7	43.8
Colectomy - Open	911	5.6	7.5	2.7	0.0	9.0	9.0	37.2
Ileostomy/Colostomy Creation, Re-siting, or Closure	294	6.0	8.0	4.0	0.0	8.0	8.0	34.7
Open Small Bowel Resection or Enterolysis	348	4.6	6.4	2.0	0.0	6.7	6.7	41.4
Anti-reflux (Nissen) - Laparoscopic	522	3.8	5.8	1.3	0.0	5.3	5.3	37.9
Appendectomy - Laparoscopic	2930	3.4	3.9	2.7	0.0	6.0	6.0	32.0
Appendectomy - Open	176	3.5	4.5	2.0	0.0	6.7	6.7	39.8
Cholecystectomy - Laparoscopic	7087	3.3	4.0	2.0	0.0	5.3	5.3	32.2
Cholecystectomy - Open	178	5.7	7.0	3.3	0.0	10.0	10.0	38.2
Minor Hernia	8166	3.6	4.5	2.0	0.0	6.0	6.0	34.7
Major Hernia	1140	4.9	6.0	3.3	0.0	6.7	6.7	27.1
Thyroidectomy	659	2.6	4.9	1.0	0.0	3.3	3.3	44.9
Hysterectomy - Abdominal	831	6.9	8.0	5.3	0.0	10.0	10.0	26.0
Hysterectomy - Laparoscopic	2643	5.2	6.2	3.3	0.0	8.0	8.0	28.6
Hysterectomy - Vaginal	1148	4.6	5.5	2.7	0.0	8.0	8.0	33.0

Carotid Endarterectomy	72	2.7	4.8	0.9	0.0	4.0	4.0	47.2
**New Procedures**								
Pancreatectomy	89	8.6	17.7	3.0	0.0	10.0	10.0	37.1
Excision of rectal tumor, transanal approach	55	3.0	4.8	0.0	0.0	6.0	6.0	54.5
Gastrorrhaphy, suture of perforated duodenal or gastric ulcer, wound, or injury	53	6.3	6.4	6.7	1.3	9.3	8.0	22.6
Laparoscopic enterolysis	49	3.0	3.6	2.0	0.0	5.0	5.0	36.7
Laparoscopic closure of enterostomy (large of small bowel) w/ resection and anastomosis)	49	4.9	5.3	4.0	0.0	10.0	10.0	38.8

A. Consumption reported in 5mg oxycodone equivalents. Some procedures have no reported current guidelines since prior prescription data was not available.

Supplementary Table 4. Kolmogorov-Smirnov analysis for opioid consumption by surgery type

**Group**	**Comparison**	**Secondary Cohort (6/1/2019 - 10/31/2021)**	
		**Kolmogorov-Smirnov Test Statistic**	***P* value**
Appendectomy	Laparoscopic vs. open	0.095	0.100
Cholecystectomy	Laparoscopic vs. open	0.212	<0.001
Colectomy	Laparoscopic vs. open	0.111	<0.001
Hernia	Major vs. minor	0.095	<0.001

Supplementary Table 5. Patient characteristics by procedure for secondary cohort

	Total	Colectomy - Laparoscopic	Colectomy - Open	Ileostomy/ Colostomy Creation, Re-siting, or Closure	Open Small Bowel Resection or Enterolysis	Anti-reflux (Nissen) - Laparoscopic	Appendectomy - Laparoscopic	Appendectomy - Open	Cholecystectomy - Laparoscopic	Cholecystectomy - Open	Minor Hernia
	N=28,721	N=1,321	N=911	N=294	N=348	N=522	N=2,930	N=176	N=7,087	N=178	N=8,166
Age, mean (SD) years											
	53.94 (16.40)	62.23 (12.57)	62.70 (13.42)	60.90 (13.31)	62.50 (14.76)	61.12 (13.32)	43.57 (17.34)	48.91 (17.49)	50.80 (17.40)	61.71 (16.29)	58.36 (15.40)
Sex											
Male	12,476 (43.4%)	611 (46.3%)	410 (45.0%)	146 (49.7%)	146 (42.0%)	138 (26.4%)	1,327 (45.3%)	96 (54.5%)	2,078 (29.3%)	96 (53.9%)	6,595 (80.8%)
Female	16,245 (56.6%)	710 (53.7%)	501 (55.0%)	148 (50.3%)	202 (58.0%)	384 (73.6%)	1,603 (54.7%)	80 (45.5%)	5,009 (70.7%)	82 (46.1%)	1,571 (19.2%)
Race/ethnicity											
White, non-Hispanic	23,054 (80.3%)	1,095 (82.9%)	780 (85.6%)	245 (83.3%)	288 (82.8%)	458 (87.7%)	2,409 (82.2%)	136 (77.3%)	5,742 (81.0%)	126 (70.8%)	6,648 (81.4%)
Black, non-Hispanic	2,573 (9.0%)	118 (8.9%)	71 (7.8%)	31 (10.5%)	35 (10.1%)	23 (4.4%)	176 (6.0%)	15 (8.5%)	581 (8.2%)	36 (20.2%)	649 (7.9%)
Hispanic	670 (2.3%)	20 (1.5%)	12 (1.3%)	5 (1.7%)	6 (1.7%)	7 (1.3%)	116 (4.0%)	7 (4.0%)	224 (3.2%)	3 (1.7%)	123 (1.5%)
Other	327 (1.1%)	8 (0.6%)	3 (0.3%)	1 (0.3%)	4 (1.1%)	4 (0.8%)	41 (1.4%)	6 (3.4%)	92 (1.3%)	4 (2.2%)	80 (1.0%)
Unknown	2,097 (7.3%)	80 (6.1%)	45 (4.9%)	12 (4.1%)	15 (4.3%)	30 (5.7%)	188 (6.4%)	12 (6.8%)	448 (6.3%)	9 (5.1%)	666 (8.2%)
Insurance type											
Private	15,280 (53.2%)	613 (46.4%)	341 (37.4%)	100 (34.0%)	136 (39.1%)	211 (40.4%)	1,882 (64.2%)	93 (52.8%)	3,781 (53.4%)	54 (30.3%)	4,046 (49.5%)
Medicare	8,105 (28.2%)	560 (42.4%)	431 (47.3%)	130 (44.2%)	166 (47.7%)	238 (45.6%)	390 (13.3%)	39 (22.2%)	1,744 (24.6%)	89 (50.0%)	2,834 (34.7%)

Medicaid	4,184 (14.6%)	106 (8.0%)	92 (10.1%)	56 (19.0%)	30 (8.6%)	56 (10.7%)	466 (15.9%)	32 (18.2%)	1,272 (17.9%)	30 (16.9%)	998 (12.2%)
Medicare and Medicaid	420 (1.5%)	22 (1.7%)	23 (2.5%)	5 (1.7%)	7 (2.0%)	10 (1.9%)	22 (0.8%)	0 (0.0%)	104 (1.5%)	3 (1.7%)	102 (1.2%)
Other/Uninsured	732 (2.5%)	20 (1.5%)	24 (2.6%)	3 (1.0%)	9 (2.6%)	7 (1.3%)	170 (5.8%)	12 (6.8%)	186 (2.6%)	2 (1.1%)	186 (2.3%)
Surgical priority											
Elective	21,775 (75.8%)	1,174 (88.9%)	439 (48.2%)	262 (89.1%)	60 (17.2%)	506 (96.9%)	221 (7.5%)	15 (8.5%)	4,643 (65.5%)	76 (42.7%)	7,853 (96.2%)
Emergent/urgent	6,946 (24.2%)	147 (11.1%)	472 (51.8%)	32 (10.9%)	288 (82.8%)	16 (3.1%)	2,709 (92.5%)	161 (91.5%)	2,444 (34.5%)	102 (57.3%)	313 (3.8%)

	**Major Hernia**	**Thyroidectomy**	**Hysterectomy - Abdominal**	**Hysterectomy - Laparoscopic**	**Hysterectomy - Vaginal**	**Carotid Endarterectomy**	**Pancreatectomy**	**Excision of rectal tumor, transanal approach**	**Gastrorrhaphy, suture of perforated duodenal or gastric ulcer, wound, or injury**	**Laparoscopic enterolysis**	**Laparoscopic closure of enterostomy (large of small bowel) w/ resection and anastomosis)**
	N=1,140	N=659	N=831	N=2,643	N=1,148	N=72	N=89	N=55	N=53	N=49	N=49
Age, mean (SD) years	57.66 (14.54)	52.36 (15.03)	49.81 (10.72)	49.42 (12.27)	51.53 (13.40)	70.86 (7.97)	64.84 (9.66)	66.98 (11.59)	56.23 (15.74)	60.16 (14.52)	57.27 (15.83)
Sex											
Male	522 (45.8%)	133 (20.2%)	0 (0.0%)	0 (0.0%)	0 (0.0%)	42 (58.3%)	48 (53.9%)	23 (41.8%)	24 (45.3%)	18 (36.7%)	23 (46.9%)
Female	618 (54.2%)	526 (79.8%)	831 (100.0%)	2,643 (100.0%)	1,148 (100.0%)	30 (41.7%)	41 (46.1%)	32 (58.2%)	29 (54.7%)	31 (63.3%)	26 (53.1%)
Race/ethnicity											
White, non-Hispanic	913 (80.1%)	475 (72.1%)	519 (62.5%)	1,977 (74.8%)	944 (82.2%)	57 (79.2%)	72 (80.9%)	48 (87.3%)	41 (77.4%)	38 (77.6%)	43 (87.8%)

Black, non-Hispanic	111 (9.7%)	94 (14.3%)	198 (23.8%)	308 (11.7%)	87 (7.6%)	9 (12.5%)	10 (11.2%)	5 (9.1%)	7 (13.2%)	5 (10.2%)	4 (8.2%)
Hispanic	33 (2.9%)	14 (2.1%)	14 (1.7%)	53 (2.0%)	31 (2.7%)	0 (0.0%)	0 (0.0%)	0 (0.0%)	0 (0.0%)	2 (4.1%)	0 (0.0%)
Other	12 (1.1%)	14 (2.1%)	17 (2.0%)	27 (1.0%)	11 (1.0%)	0 (0.0%)	2 (2.2%)	0 (0.0%)	1 (1.9%)	0 (0.0%)	0 (0.0%)
Unknown	71 (6.2%)	62 (9.4%)	83 (10.0%)	278 (10.5%)	75 (6.5%)	6 (8.3%)	5 (5.6%)	2 (3.6%)	4 (7.5%)	4 (8.2%)	2 (4.1%)
Insurance type											
Private	534 (46.8%)	361 (54.8%)	538 (64.7%)	1,780 (67.3%)	683 (59.5%)	10 (13.9%)	43 (48.3%)	22 (40.0%)	14 (26.4%)	19 (38.8%)	19 (38.8%)
Medicare	398 (34.9%)	169 (25.6%)	104 (12.5%)	383 (14.5%)	240 (20.9%)	54 (75.0%)	40 (44.9%)	32 (58.2%)	19 (35.8%)	24 (49.0%)	21 (42.9%)
Medicaid	161 (14.1%)	112 (17.0%)	149 (17.9%)	393 (14.9%)	196 (17.1%)	3 (4.2%)	5 (5.6%)	1 (1.8%)	16 (30.2%)	4 (8.2%)	6 (12.2%)
Medicare and Medicaid	28 (2.5%)	10 (1.5%)	23 (2.8%)	38 (1.4%)	12 (1.0%)	5 (6.9%)	1 (1.1%)	0 (0.0%)	1 (1.9%)	2 (4.1%)	2 (4.1%)
Other/Uninsured	19 (1.7%)	7 (1.1%)	17 (2.0%)	49 (1.9%)	17 (1.5%)	0 (0.0%)	0 (0.0%)	0 (0.0%)	3 (5.7%)	0 (0.0%)	1 (2.0%)
Surgical priority											
Elective	1,027 (90.1%)	658 (99.8%)	802 (96.5%)	2,635 (99.7%)	1,138 (99.1%)	63 (87.5%)	88 (98.9%)	54 (98.2%)	0 (0.0%)	12 (24.5%)	49 (100.0%)
Emergent/urgent	113 (9.9%)	1 (0.2%)	29 (3.5%)	8 (0.3%)	10 (0.9%)	9 (12.5%)	1 (1.1%)	1 (1.8%)	53 (100.0%)	37 (75.5%)	0 (0.0%)
